# Developing organized level of biomedical evidence: evidence-based biomedicine

**DOI:** 10.3389/fphys.2012.00428

**Published:** 2012-11-08

**Authors:** Fakher Rahim, Akbar Soltani, Vahid Haghpanah

**Affiliations:** Endocrinology and Metabolism Research Center, Tehran University of Medical SciencesTehran, Iran

Evidence-Based Medicine (EBM) is a methodological and systematic approach, which is considered as the most robust form of the empirical medicine as it sorts evidence according to their credibility. It is widely used in clinical decision-making and has several advantages, including: (1) provision of a very well-founded and objective method to adhere to high quality and safety standards in medical practice, (2) facilitation of translation of clinical research findings into clinical practice, and (3) significant reduction of health care costs. EBM can be summarized into five practical steps include searching for an answer to a clinical question, searching for the evidence, critically appraising and reviewing the existing evidence, combining credible evidence with clinical experience and the patients' viewpoints, and finally, evaluating the process.

As it is widely believed that evidence is not properly classified and there is no credible assessment method in data on biomedical literatures, it could be suggested that EBM is of limited use in this branch of science (Clancy and Cronin, 2005). Therefore, in biomedicine, although there are literature redundancies and large number of evidences, they are only useful if a systematic searching approach is taken. Moreover, a structured process needs to be in place to analyze the findings and critically appraise them.

Due to the existence of overwhelmingly disorganized data in the field of biomedicine that lead to non-categorized and non-screened information, it could be suggested that there is a need for development of a novel methodology considering different levels of evidence (Ioannidis, 2005; Cho, 2007). Moreover, the hierarchy of EBM in biomedicine is questionable; the need is more highlighted (Mantzoukas, 2008).

Evidence Based Bio-Medicine (EBBM) can be considered as a novel method in biomedical literatures. This technique focuses on the evaluation and ranking of different articles with widely available techniques in the field based on their credibility, limitations, and biases. The aim of this letter is to analyze the effectiveness of EBBM in order to shed some light on its limitations. It could lead to classification of evidence on biomedical science issues and facilitate their practical application with the view of addressing the raised issue in this letter.

In order to present the case more clearly, the following examples are used:

1. Various studies have been carried out in order to shed some light on the genetic susceptibility to thyroid cancer using molecular diagnostic and predictive biomarker (Ruggeri et al., 2008; Nikiforova and Nikiforov, 2009; Landa and Robledo, 2011). The methods and techniques used in different studies may vary. Sample size and selection methods, using fresh tissue samples or paraffin embedded blocks and especially, the details of techniques used in different studies can differ. This can raise the question as to whether there exists a method of classification, way of evaluation, assessment of scientific credibility, and ultimately, a ranking of the level of evidence on their validity. Moreover, it should be clarified that how other researchers interested in complementary studies on the same subject can conduct their study and select the most valid studies based on what criteria?
2. There is enormous number of meta-analyses of genome-wide association studies (GWAS) that aimed to detect genetic variants with gradually smaller effects, but force to publish the outcomes of the studies of new genetic associations has narrowed the time available for watchful consideration of all of their methodological aspects. A survey of the literature from 2007 to 2010, to offer empirical evidence on the methods used in meta-analyses of GWAS, showed that a great variety of methods are being used, but the logic of their selection is often unclear (Gögele et al., 2012). This review also highlighted how important methodological features have achieved unsatisfactory attention, potentially leading to missed opportunities for improving gene discovery and characterization. Evaluation of power to replicate findings was inadequate, and the number of variants selected for replication was not associated with replication sample size. Additional methodological efforts and clear guidance are required to offer the optimal methods or trade-offs between alternative methods.

Finally, it could be suggested that different levels of evidence should be based on: (1) the study types such as *in vitro, in vivo*, and clinical trial (2) laboratory techniques used (3) “omics” approach to the hypothesis testing like genomics, proteomics, and (4) Utilize a checklist to classify and grade the evidence in biomedicine. Moreover, further investigation and testing of the validity of these tools is needed, which indicate that the EBBM scales are valid and reliable instruments for measuring biomedical datas' confidence in the process and the outcomes basing on the evidence.

Hopefully, this may encourage scientists in the field of biomedicine, molecular epidemiology, bioinformatics, and translational medicine to contribute in the foundation of a novel and functional methodological approach using different levels of evidence raised above.
